# Complexities of trial recruitment in the care home setting: an illustration via the DCM™epic (dementia care mapping™: to enable person-centred care in care homes) trial

**DOI:** 10.1186/1745-6215-16-S2-P96

**Published:** 2015-11-16

**Authors:** Claire Surr, Amanda Lilley-Kelly, Liz Graham, Rebecca Walwyn, Robert Cicero, Alys Griffiths, Byron Creese, Lucy Garrod

**Affiliations:** 1 University of Leeds, Leeds, UK; 2 Leeds Beckett University, Leeds, UK; 3 Kings College London, London, UK; 4 Oxford Health NHS Foundation Trust, Oxford, UK

## 

Conducting trials in care homes is complex on multiple levels. Here we focus on recruitment issues surrounding: a) care home selection to participate in research, b) selection and involvement of participants fulfilling various roles (residents, relatives, staff), c) consent in the context of the Mental Capacity Act, and d) scheduling researcher time to undertake complex recruitment processes across multiple care homes.

To ensure generalizability of results, EPIC care homes were selected to form a stratified random sample of a known sampling frame. This was done by first defining catchment areas around each of three participating UK hubs. Randomly ordered listings of all eligible care homes within those areas were then produced, with batches of care homes sent trial information and followed up by researchers.

Trial participation for a home requires agreement to take part from residents and their relatives (as personal consultees and providers of proxy data), as well as staff involvement to provide data (proxy and self) and be trained to deliver the DCM™ intervention. This requires complex, lengthy discussions with all parties, provision of tailored information sheets specific to intended role and capacity, and involvement of trial experts to explain DCM™ in more detail to staff.

The trial aims to recruit 50 care homes (750 residents) by the end of 2015. Thus there is the need to balance the complexity of processes with required speed of recruitment - a task which is achieved by detailed monitoring of projected researcher workload in relation to care home commitments and availability.

